# General Preparation of Nickel-Based Compound/Carbon Nanotube Catalysts for the Urea Oxidation Reaction

**DOI:** 10.3390/nano16191276

**Published:** 2026-10-08

**Authors:** Yanjie Xia, Sheng-Zhi Ma, Yi-Jun Chen, Jia-Wen Du, Chao Zhang, Haibin Sun, Ze-Xing Cai

**Affiliations:** 1 Collaboration Innovative Center of Henan Province for Energy-Saving Building Materials, Xinyang Normal University, Xinyang 464000, China; yj_xia_2026@xynu.edu.cn; 2 College of Physics and Electronic Engineering, Xinyang Normal University, Xinyang 464000, China; msz020809@xynu.edu.cn (S.-Z.M.); chen.yijun@xynu.edu.cn (Y.-J.C.); djw0529@xynu.edu.cn (J.-W.D.); zhangc@xynu.edu.cn (C.Z.); sunhaibin@xynu.edu.cn (H.S.)

**Keywords:** HOFs, template, nanotubes, electrocatalyst, urea oxidation reaction

## Abstract

Leveraging the flexibility and adaptability of hydrogen bonding, hydrogen-bonded organic framework (HOF) nanowires can serve as effective templates and precursors for the synthesis of hollow structures composed of uniform multielement compounds. Benefiting from the mesoporous nanotube structure and synergistic active sites, the fabricated Ru-Ni_2_P/C catalyst delivers low potential, high current density, and good stability. When employed for urea electrolysis, the catalyst enables a current density of 10 mA cm^−2^ at potentials of 1.36 V vs. RHE. These findings offer a promising structural and compositional design strategy for developing high-performance electrocatalysts.

## 1. Introduction

As economic expansion and industrialization accelerate globally, energy scarcity has become an increasingly prominent concern worldwide [1]. The exploration of novel environmentally friendly energy materials and the advancement of associated technologies represent an urgent priority for achieving long-term sustainability. Water electrolysis is emerging as a promising alternative for clean hydrogen production in sustainable manner [2]. Compared with the oxygen evolution reaction (OER, 1.23 V vs. RHE), the urea oxidation reaction (UOR) proceeds at a substantially lower anodic potential (0.37 V vs. RHE) [3,4]. Furthermore, as a waste byproduct, the extensive presence of urea in industrial and human wastewater can permeate both groundwater systems and the atmosphere, thereby engendering public health concerns. Therefore, the simultaneous treatment of a potentially dangerous environmental waste and the production of a valuable fuel can be achieved through urea electrooxidation.

Nickel-based materials have emerged as economical substitutes for noble metal catalysts in urea electrooxidation [5,6]. Recent findings show that nickel-based compounds, known to catalyze reactions such as water splitting [7], hydrazine oxidation [8], and ethanol oxidation [9,10], hold great promise as catalysts for urea oxidation because of their unique layered structures [11]. The incorporation of secondary metal elements into nickel phosphide has been established to mitigate the overpotential required for urea oxidation and enhance long-term stability [12]. Furthermore, nanostructures with a high specific surface area facilitate efficient mass transport and expose more active sites. Therefore, it is highly desirable to establish a facile method for constructing high-surface-area nanostructures based on composition-tunable nickel-based compounds to enhance electrocatalytic UOR performance.

In this work, a series of nickel-based compound/C nanotubes were generally synthesized via a hydrothermal strategy using modified HOFs nanowires as sacrificial templates [13]. Upon reaction with Ni^2+^ and phosphoric acid (or selenious acid, thiourea (TU), etc.) species, HOF nanowires are converted into Ni-BTC(P)/HOF (or Ni-BTC(Se)/HOF, Ni-BTC(S)/HOF, etc., where BTC stands for benzene-1,3,5-tricarboxylic acid) nanotubes by virtue of the flexibility and adaptability of hydrogen bonds. Hybrid nanotubes containing nickel-based compound and N-doped carbon were obtained through a carbonization process. Benefiting from the mesoporous nanotube structure and synergistic active sites, the calcined nickel phosphide catalyst (Ru-Ni_2_P/C) possessed superior activity towards UOR (285 mA·cm^−2^ at 1.6 V versus reversible hydrogen electrode) and long-term durability for over 80 h at a current density of 10 mA cm^−2^. These results highlight the potential of nickel phosphide nanotube-based porous architectures for UOR. This work presents a promising HOF-templated strategy for the fabrication of porous electrocatalysts toward efficient UOR.

## 2. Results and Discussion

**Figure 1:**
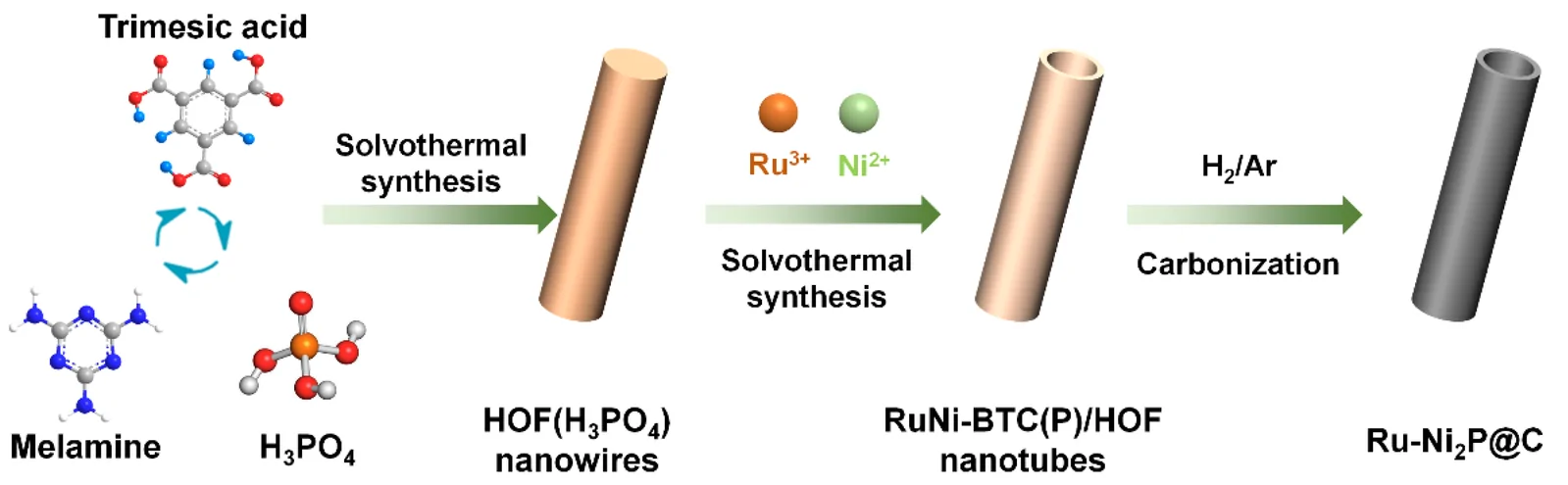
Schematic illustration of the synthesis of mesoporous Ru-Ni_2_P@C nanotubes.

As illustrated in Figure 1, Ru-Ni_2_P/C (and NiPO_x_/C) nanotubes were fabricated via a template-assisted strategy using one-dimensional HOF nanowires as the sacrificial scaffold. In the first step, the hydrogen-bonded organic framework (HOF(H_3_PO_5_)) template is synthesized through the assembly of BTC, MA (melamine), and H_3_PO_4_ in methanol at 150 °C (Figure S1). Next, these HOF(H_3_PO_5_) nanowires were subjected to react with Ru^3+^, Ni^2+^ ions at 150 °C to generate the RuNi-BTC(P)/HOF nanotubes. During this process, the HOF template was gradually consumed and tubular RuNi-BTC(P)/HOF (and Ni-BTC(P)/HOF) intermediates were formed (Figures S2 and S3). The balance between the affinities of Ni^2+^/Ru^3+^ for BTC and H_3_PO_4_ is essential for maintaining the original morphology and promoting the formation of MOF nanotubes [14]. To eliminate residual organic components and enhance electrical conductivity, the as-prepared sample was subjected to thermal annealing at 500 °C for 2 h under a mixed 5% H_2_/Ar atmosphere and the Ru-Ni_2_P/C (and NiPO_x_/C) nanotube catalysts were obtained (Figures S4 and S5). This versatile method can be readily extended to the synthesis of Ni_9_S_8_/C, Ru–NiS/Ni_9_S_8_/C, Ni_3_Se_4_/C, and Ru–Ni_3_Se_4_/C nanotube catalysts, highlighting its broad applicability (Figures S6–S11).

**Figure 2:**
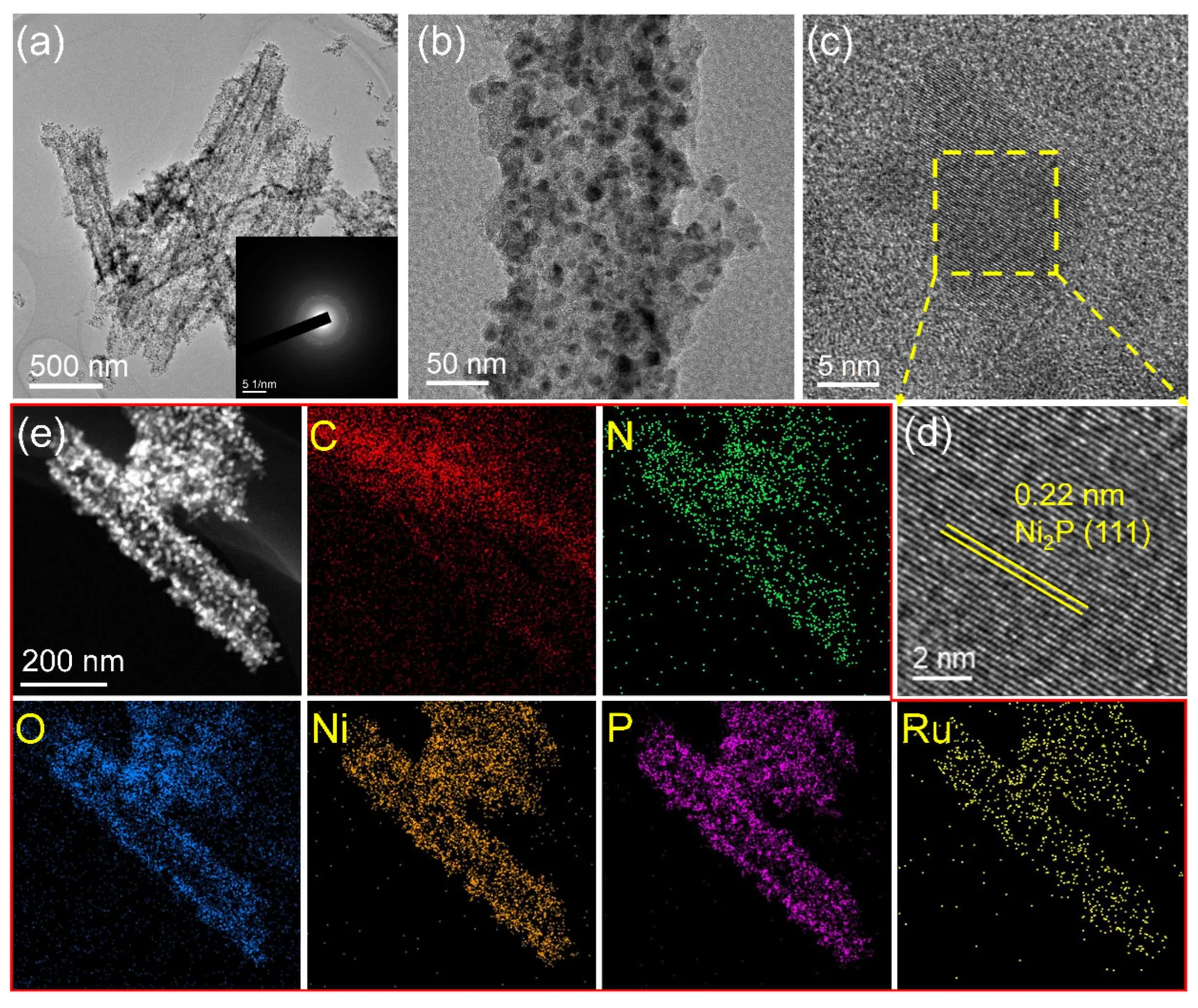
(**a**,**b**) TEM images and selected area electron diffraction (SAED) patterns (inset in a) of Ru-Ni_2_P/C nanotubes, (**c**,**d**) high-resolution TEM images of Ru-Ni_2_P/C nanotubes, (**e**) HAADF-STEM image of Ru-Ni_2_P/C nanotubes and the elemental mapping images of C, N, O, Ni, P, and Ru (in red box).

The morphology and microstructure of the as-prepared Ru-Ni_2_P/C nanotubes were further characterized by transmission electron microscopy (TEM). As shown in Figure 2a,b, the tubular morphology was well preserved during the pyrolysis process. Meanwhile, The SAED pattern (inset of Figure 2a) can be indexed to the crystal phase of Ni_2_P, and no diffraction rings corresponding to metallic Ru are observed. Accordingly, we can reasonably infer that the Ni, Ru, and phosphorus species from the precursor were converted into Ru-doped Ni_2_P nanocrystals (inset in Figure 2a), which were firmly anchored to nitrogen-doped carbon nanotubes generated through carbonization of the MA and BTC organic linkers. The high-resolution TEM images (Figure 2c,d) reveal clear lattice fringes with a spacing of ~0.22 nm, corresponding to the (111) planes of Ni_2_P. Furthermore, the HAADF-STEM (High Angle Angular Dark Field-Scanning Transmission Electron Microscopy) image and corresponding elemental mapping results (Figure 2e) demonstrate that C, N, O, P, Ni, and Ru are homogeneously distributed throughout the entire 1D nanostructure without obvious elemental segregation. This morphology facilitates the separation of alloy particles, exposing more active sites and thus improving the electrocatalytic UOR activity and long-term catalytic durability.

**Figure 3:**
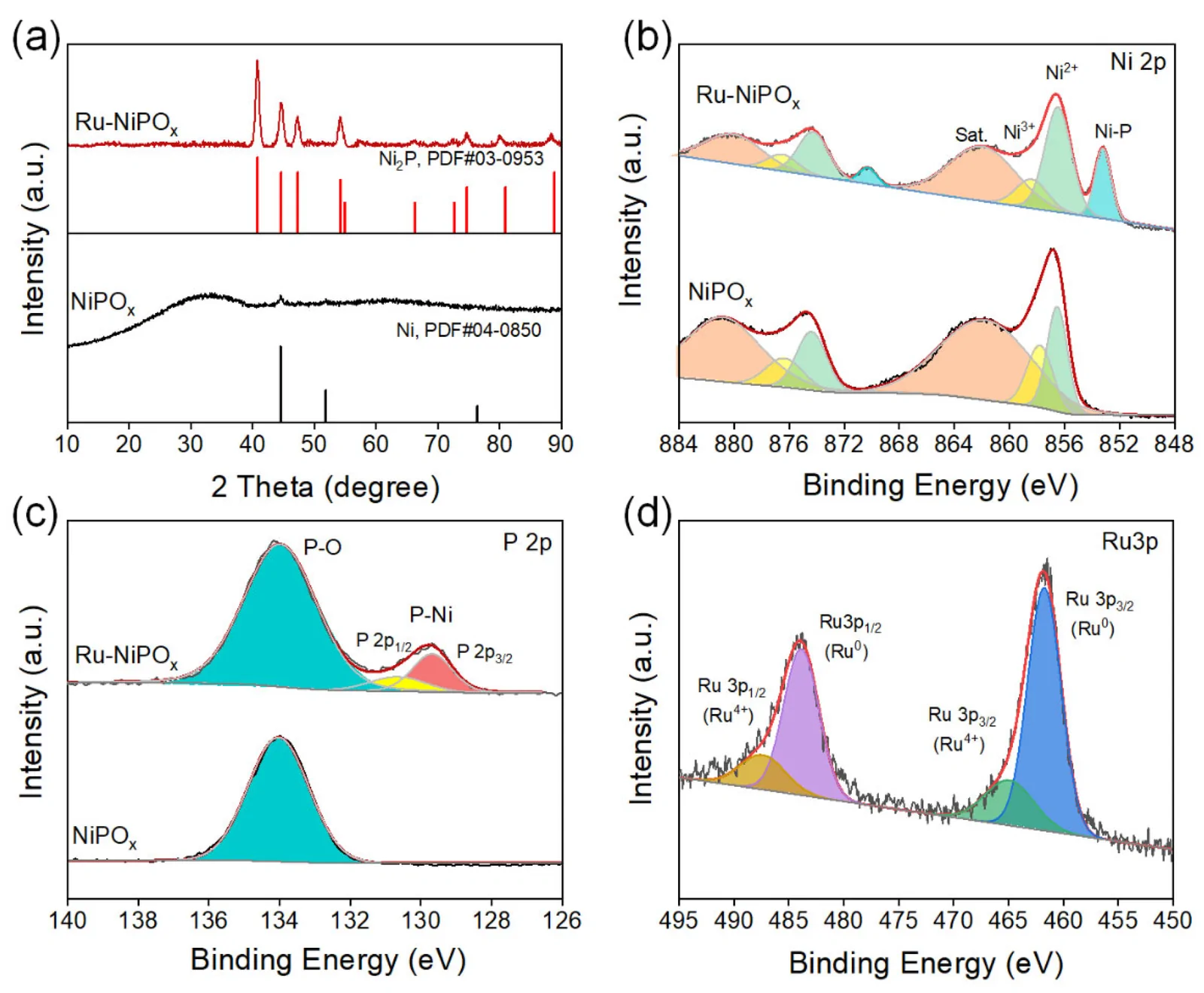
(**a**) XRD patterns of NiPO_x_/C and Ru-Ni_2_P/C. High-resolution XPS spectra corresponding to (**b**) Ni 2p, (**c**) P 2p, and (**d**) Ru 3p of NiPO_x_/C and Ru-Ni_2_P/C.

The crystal structures of the catalysts were further studied using X-ray diffraction (XRD) analysis. As shown in Figure 3a, the pristine NiPO_x_/C exhibits a broad diffraction feature, indicating its predominantly amorphous nature. After Ru incorporation, several weak diffraction peaks emerge, which can be indexed to the Ni_2_P phase (PDF#03-0953) [15], suggesting the partial phosphidation of the phosphate framework during the reductive calcination process.

The surface electronic structures of NiPO_x_/C and Ru–Ni_2_P/C were analyzed by X-ray photoelectron spectroscopy (XPS). The high-resolution Ni 2p spectrum of Ru–Ni_2_P/C (Figure 3b) contains Ni–P (853.2 eV), Ni^2+^ (856.5 eV), Ni^3+^ (858.4 eV), and satellite peaks,^11^ whereas NiPO_x_/C shows only Ni^2+^ (856.5 eV) and Ni^3+^ (857.8 eV) species associated with nickel phosphate. The metallic Ni–P component in Ru–NiPO_x_/C imply that the reductive atmosphere and Ru incorporation facilitate the partial reduction in Ni species and the formation of metal phosphide domains [16]. The P 2p spectrum (Figure 3c) of Ru–Ni_2_P/C contains dominant P–O (134.0 eV) species together with additional P–Ni (130.6 eV, 129.7 eV) components at lower binding energies, further confirming the coexistence of phosphate and phosphide species after calcination [17]. This result is consistent with the XRD analysis, indicating incomplete phosphidation and the preservation of abundant phosphate species within the framework. The Ru 3p spectrum (Figure 3d) reveals the coexistence of metallic Ru^0^ and oxidized Ru^4+^ species [18]. Such mixed-valence Ru species are expected to induce strong electronic interactions with the NiPO_x_/C matrix, thereby modulating the local electronic structure and generating abundant interfacial active sites.

**Figure 4:**
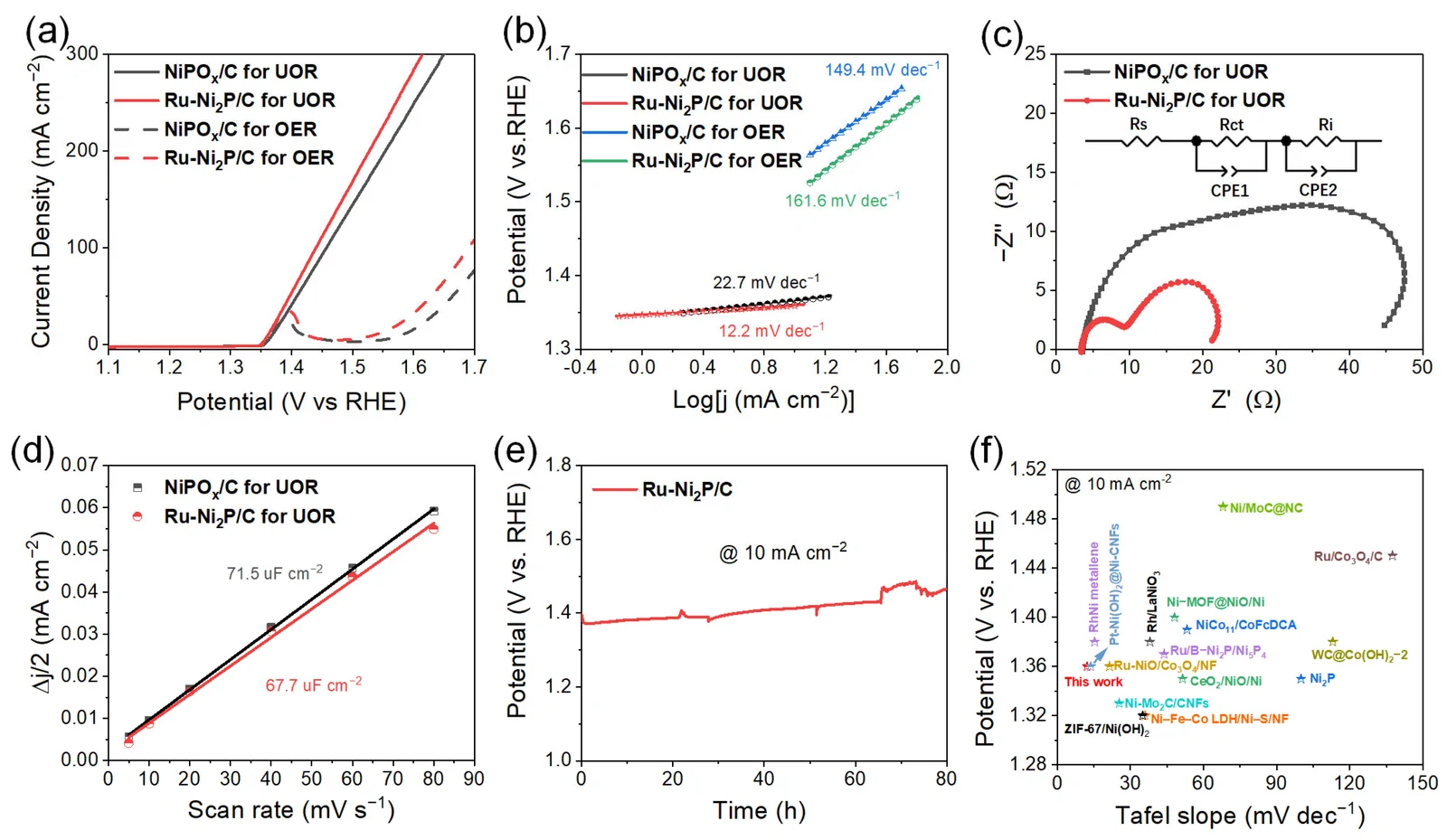
(**a**) LSV curves, and (**b**) Tafel plots of NiPO_x_/C and Ru–Ni_2_P/C catalysts in 1 M KOH aqueous electrolyte with and without 0.33 M urea. (**c**) Nyquist plots tested at 1.347 V (vs. RHE), and (**d**) C_dl_ values. of NiPO_x_/C and Ru–Ni_2_P/C catalysts, (**e**) chronopotentiometry test of Ru–Ni_2_P/C at 10 mA cm^−2^, (**f**) Comparison of the potential and Tafel plots of Ru-Ni_2_P/C with recently reported UOR catalysts (See Table S1).

The electrocatalytic performances of NiPO_x_/C and Ru–Ni_2_P/C were subsequently evaluated in a standard three-electrode configuration (Figure 4). OER and UOR polarization curves were recorded by linear sweep voltammetry (LSV) in 1 M KOH and 1 M KOH supplemented with 0.33 M urea, respectively. As illustrated in Figure 4a, NiPO_x_/C and Ru–Ni_2_P/C delivered a current density of 10 mA cm^−2^ at potentials of 1.36 and 1.37 V vs. RHE, respectively, for the UOR, which are 160 and 200 mV lower than those required for the OER (Figure 4a). These results indicate that the UOR proceeds more readily than the OER, requiring a substantially lower operating potential. Moreover, Ru–Ni_2_P/C required a potential that was 10 mV lower than that of NiPO_x_/C to achieve 10 mA cm^−2^ for the UOR, indicating that Ru-induced electronic modulation effectively enhances the intrinsic catalytic activity of the Ni active sites.

The reaction kinetics of the UOR and OER on NiPO_x_/C and Ru–Ni_2_P/C were further evaluated using Tafel analysis and electrochemical impedance spectroscopy (EIS). As presented in Figure 4b, the UOR Tafel slopes of NiPO_x_/C and Ru–Ni_2_P/C were determined to be approximately 22.7 and 12.2 mV dec^−1^, respectively, both of which are substantially lower than their corresponding OER values, suggesting more favorable UOR kinetics. Notably, the smaller Tafel slope of Ru–Ni_2_P/C relative to NiPO_x_/C indicates more rapid reaction kinetics and highlights the beneficial role of Ru incorporation in accelerating the UOR process. The substantial variation in the Tafel slopes may suggest that the two catalysts follow distinct rate-determining steps (RDSs). The charge-transfer resistance (R_ct_) derived from EIS measurements also provides insight into the UOR kinetics at the catalyst–electrolyte interface. Ru–Ni_2_P/C performs the lower charge transfer resistance of 18.1 Ω lower than NiPO_x_/C (30.3 Ω), indicating more efficient interfacial charge transfer and faster UOR kinetics. This observation is consistent with the results derived from the Tafel slope analysis. The UOR performance of Ni_9_S_8_/C, Ru–NiS/Ni_9_S_8_/C, Ni_3_Se_4_/C, and Ru–Ni_3_Se_4_/C nanotube catalysts were also conducted and presented in Figure S13. Notably, Ru–Ni_2_P/C exhibits the highest catalytic activity and the fastest reaction kinetics among all the prepared catalysts. The enhanced UOR performance of Ru–Ni_2_P/C nanotubes originates from the synergistic interplay between electronic structure regulation and the favorable nanotubular architecture.

The electrochemical double-layer capacitances (C_dl_) of NiPO_x_/C and Ru–Ni_2_P/C were estimated by analyzing the non-Faradaic capacitive currents obtained from CV measurements at different scan rates (Figure S14). The charging current density (ic) was plotted against the scan rate (ν), and the corresponding linear fitting curves were obtained (Figure 4d). The C_dl_ values, obtained from the slopes of the fitted linear plots, were determined to be 67.7 and 71.5 μF cm^−2^ for NiPO_x_/C and Ru–Ni_2_P/C, respectively. Despite its lower C_dl_ and thus potentially smaller electrochemically accessible surface area, Ru–Ni_2_P/C delivered superior OER performance. This result suggests that its enhanced electrocatalytic behavior mainly originates from higher intrinsic activity rather than a larger number of exposed active sites. Electrochemical stability is also essential for evaluating the long-term UOR performance of electrocatalysts. A chronopotentiometric test was performed at a constant current density of 10 mA cm^−2^, during which the operating potential remained stable at approximately 1.40 V over 80 h with slight degradation (Figure 4e). The lower potential of 1.36 V at 10 mA cm^−2^ and tafel slope obtained from Ru–Ni_2_P/C was comparable or superior to those of the recently reported cutting-edge UOR catalysts (Figure 4f and Table S1).

## 3. Conclusions

In summary, this study demonstrates the versatile use of HOF nanowires as self-sacrificial templates for constructing nickel-based compound/C nanotubes with porous hierarchical one-dimensional architecture. Such structural features are expected to provide abundant exposed active sites, a large specific surface area, and efficient mass and electron transport, thereby enhancing the electrocatalytic performance. The mesoporous nanotubular architecture and synergistic active sites endow Ru–Ni_2_P/C with a low operating potential, high current density, and excellent stability. This work established a versatile route to multicomponent MOF precursors incorporating both organic and inorganic ligands for the development of high-performance energy conversion and storage materials.

## Data Availability

The original contributions presented in this study are included in the article and Supplementary Materials. Further inquiries can be directed to the corresponding author.

## Supplementary Materials

The following supporting information can be downloaded at: https://www.mdpi.com/article/10.3390/nano16191276/s1, Experimental; Figure S1: SEM of MA-BTC(H_3_PO_4_) nanowires at different magnifications; Figure S2: SEM of (a,b) Ni-BTC(P)/HOF, and (c,d) RuNi-BTC(P)/HOF nanotubes at different magnifications; Figure S3: XRD patterns of the NiPO_4_/HOF and Ru-NiPO4/HOF; Figure S4: SEM of (a,b) NiPOx/C, and (c,d) Ru-Ni_2_P/C nanotubes at different magnifications; Figure S5: (a,b) TEM images, (c) HRTEM image, and SAED pattern (inset) of NiPOx/C. (d) STEM image and the corresponding elemental mappings of NiPO_x_/C; Figure S6: SEM of (a,b) Ni-BTC(S)/HOF, and (c,d) RuNi-BTC(S)/HOF nanotubes at different magnifications; Figure S7: SEM of (a,b) NiS/Ni_9_S_8_/C, and (c,d) Ru-Ni_9_Se_8_/C nanotubes at different magnifications; Figure S8: XRD patterns of NiS/Ni_9_S_8_/C and Ru-Ni_9_S_8_/C catalysts; Figure S9: SEM of (a,b) Ni-BTC(Se)/HOF, and (c,d) RuNi-BTC(Se)/HOF nanotubes at different magnifications; Figure S10: SEM of (a,b) Ni_3_Se_4_/C, and (c,d) Ru-Ni_3_Se_4_/C nanotubes at different magnifications; Figure S11. XRD patterns of Ni_3_Se_4_/C and Ru-Ni_3_Se_4_/C catalysts; Figure S12: High-resolution XPS spectra corresponding to (a) N 1s, and (b) O 1s; Figure S13: (a) LSV curves, (b) Tafel plots, and (c) Nyquist plots tested at 1.347 V (vs. RHE) of NiS/Ni_9_S_8_/C and Ru–Ni_9_S_8_/C catalysts in 1M KOH aqueous electrolyte with and without 0.33 M urea; (d). (a) LSV curves, (b) Tafel plots, and (c) Nyquist plots tested at 1.347 V (vs. RHE) of Ni_3_Se_4_/C and Ru–Ni_3_Se_4_/C catalysts in 1M KOH aqueous electrolyte with and without 0.33 M urea; Figure S14: CV curves of (a) NiPO_x_/C, (b) Ru-Ni_2_P/C at different scan rates (5, 10, 20, 40, 60, and 80 mV/s); Table S1: Comparison of catalytic performance of NiPO_x_/C, Ru-Ni_2_P/C and some recently reported catalysts toward UOR; References [19,20] are cited in the Supplementary Materials.
