# Supplementary material for: General Preparation of Nickel-Based Compound/Carbon Nanotube Catalysts for the Urea Oxidation Reaction
Source: Nanomaterials (Basel). 2026 Oct 8;16(19):1276. doi: 10.3390/nano16191276 (PMC13667290; doi:10.3390/nano16191276)
Supplement: Supplementary file 1 [file nanomaterials-16-01276-s001.zip › nanomaterials-4597839-supplementary.pdf]

General Preparation of Nickel-Based Compound/Carbon Nanotube Catalysts for the Urea Oxidation Reaction

Yanjie Xia [1], Sheng-Zhi Ma [2], Yi-Jun Chen [2], Jia-Wen Du [2], Chao Zhang [2], Haibin Sun[2] and Ze-Xing Cai [1,2,*]

1 Collaboration Innovative Center of Henan Province for Energy-Saving Building Materials, Xinyang Normal University, Xinyang 464000, China; yj_xia_2026@xynu.edu.cn

2 College of Physics and Electronic Engineering, Xinyang Normal University, Xinyang 464000, China; msz020809@xynu.edu.cn (S.-Z.M.); chen.yijun@xynu.edu.cn (Y.-J.C.); djw0529@xynu.edu.cn (J.-W.D.); zhangc@xynu.edu.cn (C.Z.); sunhaibin@xynu.edu.cn (H.S.)

* Correspondence: caizexing.2009@163.com.

Experimental

2.1. Reagents

All solvents and chemicals were of analytical grade and used as received. 1.3.5-Benzenetricarboxylic acid (BTC, 95%), melamine (MA, ≥ 99.0%), and urea were obtained from Sigma-Aldrich Chemical Reagent Co., Ltd. Nickel acetate tetrahydrate (99.0%) were obtained from Fujifilm Wako Pure Chemical Corporation. Methanol (99.5% purity) was purchased from Shanghai Titan Scientific Co., Ltd. A Nafion-perfluorinated resin solution (5 wt. %) and KOH (≥ 85.0%) were purchased from Macklin. Hydrophilic carbon fiber paper was purchased from Toray Industries, Inc., and deionized water (resistivity: ≥18.25 MΩ·cm) was obtained using an ultrapure water system (ULUR-IV-10T; ULUPURE).

2.2. Synthesis of the nanotubes of nickel phosphate

In a typical synthesis, 1.2 mmol of benzene-1,3,5-tricarboxylic acid (BTC), 1.8 mmol of melamine (MA), 33 ml of methanol, and 1.8 mmol of H_3PO_4 were added to a 50 ml vial. After sealed, the mixture was sonicated for 2 hours until a homogeneous milk-like suspension formed. The suspension was transferred to a Teflon-lined stainless-steel autoclave and heated to 150 °C, where it was maintained for 12 h and then air-cooled to room temperature. Secondly, 1.8 mmol of nickel nitrate ($Ni(NO_3)_2·6H_2O$) and 0.15 mmol of $RuCl_3$ were added into the as-obtained suspension and stirred for 20 minutes. Next, the mixture was sealed again into the stainless-steel autoclave and heated to 150 °C for another 24 h. The obtained precipitate was centrifuged and washed for several times with absolute ethanol to remove the remaining reactants. The resulted product was dried at 60 °C for 12 h to obtain the nanotubes of nickel phosphate (RuNi-BTC(P)/HOF nanotubes). For comparison, Ni-BTC(P)/HOF nanotubes was prepared similarly in the absence of $RuCl_3$.

The dried product obtained above was placed at the bottom of a ceramic boat, which was then placed in a tube furnace and calcined at 500 °C for 2 hours with a heating rate of 5 °C/min, under a 5% H_2/Ar gas atmosphere. Ru-Ni_2P/C and $NiPO_x$/C cataltsts were obtained.

NiS/Ni_9S_8/C, Ru-Ni_9S_8/C, Ni_3Se_4/C, and Ru-Ni_3Se_4/C nanotubes were also

prepared using above mentioned solvothermal method, except for replacing phosphoric acid with thiourea and selenous acid.

2.4 Structural Characterization

Crystal structures of the samples were characterized by X-ray diffraction (XRD) with Cu-Kα radiation (40 kV, 40 mA, λ = 1.5418 Å; Rigaku SmartLab9 instrument). Morphologies of the samples were investigated using a field-emission scanning electron microscope (FESEM) (Hitachi S-4800) operated at an accelerating voltage of 15 kV and a transmission electron microscope (TEM; JEM-ARM200F "NEO ARM," JEOL) equipped with aberration correctors for image- and probe-forming lens systems (CEOS GmbH) and an energy-dispersive X-ray spectrometer (JED-2300T, JEOL) operated at an accelerating voltage of 200 kV. X-ray photoelectron spectroscopy (XPS) measurements were performed on an XPS instrument (K-ALPHA 0.5EV) with a monochromatic Al Kα X-ray source.

2.5 Electrochemical Measurements

All the electrochemical measurements were performed in a three-electrode system using an electrochemical workstation (CHI 760E, Shanghai Chenhua, China). A homogeneous ink was prepared by dispersing the sample (8 mg) and Nafion solution (20 μL) in a water/ethanol solution (800 μL) with a volume ratio of 1:3. After ultrasonic treatment for more than 60 min, 10 μL of the as-prepared ink was loaded onto a 2 mm × 20 mm piece of carbon paper (the loading area was controlled to be 2 mm × 10 mm, loading ca. 0.50 $mg{\cdot}cm^{-2}$). After drying, the carbon paper was transferred to a holder as the working electrode. A graphite rod and Hg/HgO electrode (0.1053 V versus RHE) served as the counter and reference electrodes, respectively. The linear sweep voltammograms (LSV) were measured at a scan rate of 5 $mV{\cdot}s^{-1}$ in an Ar-saturated 1 M KOH/0.33M urea solution. All measured potentials were calibrated to a reversible hydrogen electrode (RHE) using the following equation: $E_{RHE} = E_{SCE} + (0.1053 + 0.059 \times pH)$ [51]. All the data were collected without iR compensation. To obtain the Tafel plot, the polarization curves were plotted as overpotential versus log current density, and the linear portions at low overpotentials were fitted to the Tafel equation ($\eta = a + b\log j$, where η is the overpotential, b is the Tafel slope, j is the current density, and a is the

exchange current density). Electrochemical impedance spectroscopy (EIS) measurements were conducted in the potentiostatic mode in the frequency range of 1 MHz to 0.1 Hz, with an amplitude of 10 mV. Cyclic voltammetry curves in the region 1.100-1.200 V (vs. RHE) were measured at different scan rates. The electrochemically active surface area (ECSA) could be evaluated from the slope of the plot of the charging current versus scan rate, which was proportional to the double-layer capacitance (C_{dl}, ESCA = C_{dl}/C_s, $C_s = 40\ \mu F\ cm^{-2}$) [19, 20]. The long-term stabilities of the catalysts were tested by chronopotentiometry.

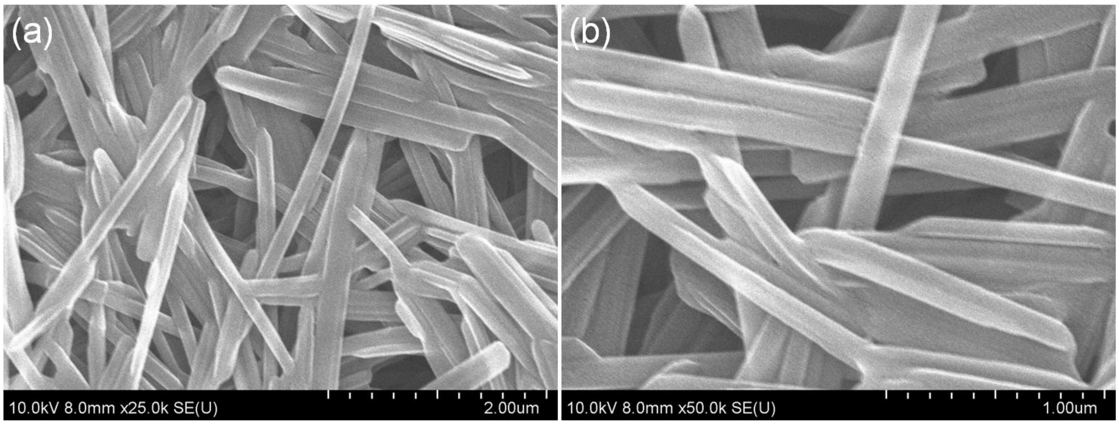

Figure S1. SEM of MA-BTC(H_3PO_4) nanowires at different magnifications.

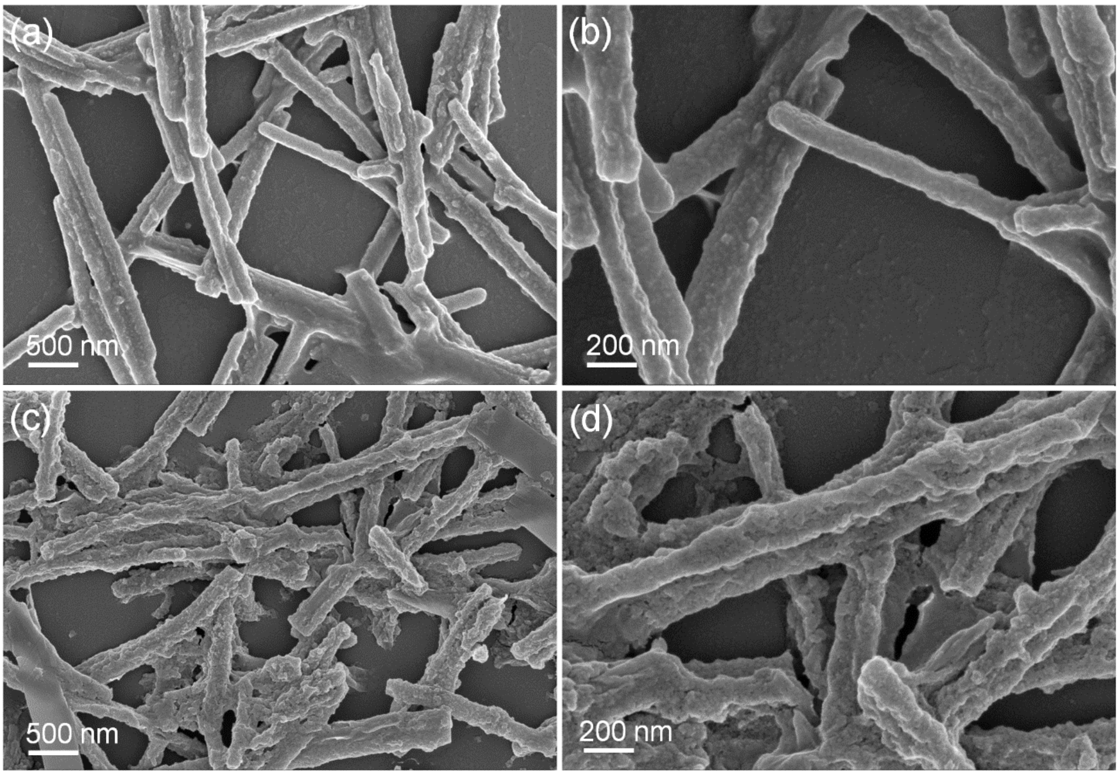

Figure S2. SEM of (a-b) Ni-BTC(P)/HOF, and (c-d) RuNi-BTC(P)/HOF nanotubes at different magnifications.

SEM images revealed that the morphology of the Ni-BTC(P)/HOF and RuNi-BTC(P)/HOF nanotubes was affected slightly by the Ru^{3+} (Figure S2). When no $RuCl_3$ added, Ni-BTC(P)/HOF nanotubes with smooth surfaces were obtained (Figure S2a,b). However, the surface of nanotubes presented a significant coarsening phenomenon when the $RuCl_3$ was added (Figure S2c,d), which is thought to be caused by the stronger bonding effect between Ru^{3+} and phosphate (PO_4^{3+}).

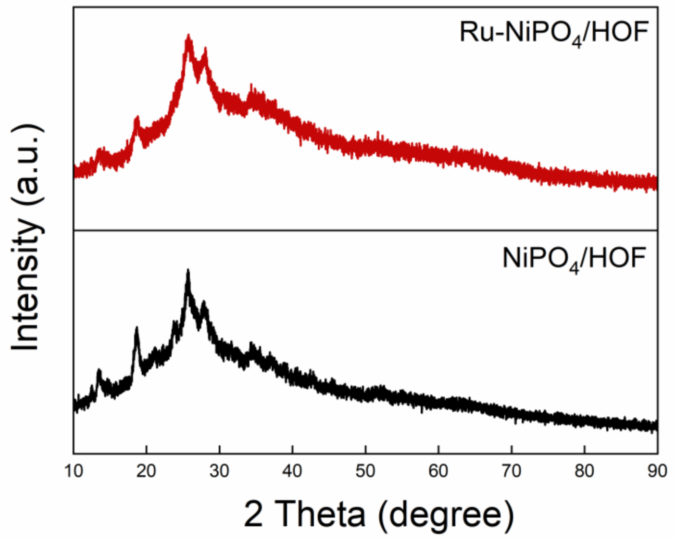

Figure S3. XRD patterns of the $NiPO_4$/HOF and Ru-$NiPO_4$/HOF.

XRD results of $NiPO_4$/HOF and Ru-$NiPO_4$/HOF showed that the HOF phase still persists after the solvothermal reaction (Figure S3). Except for the diffraction peaks corresponding to the HOF phase, no diffraction peaks attributable to $NiPO_4$ were detected, indicating its poor crystallinity.

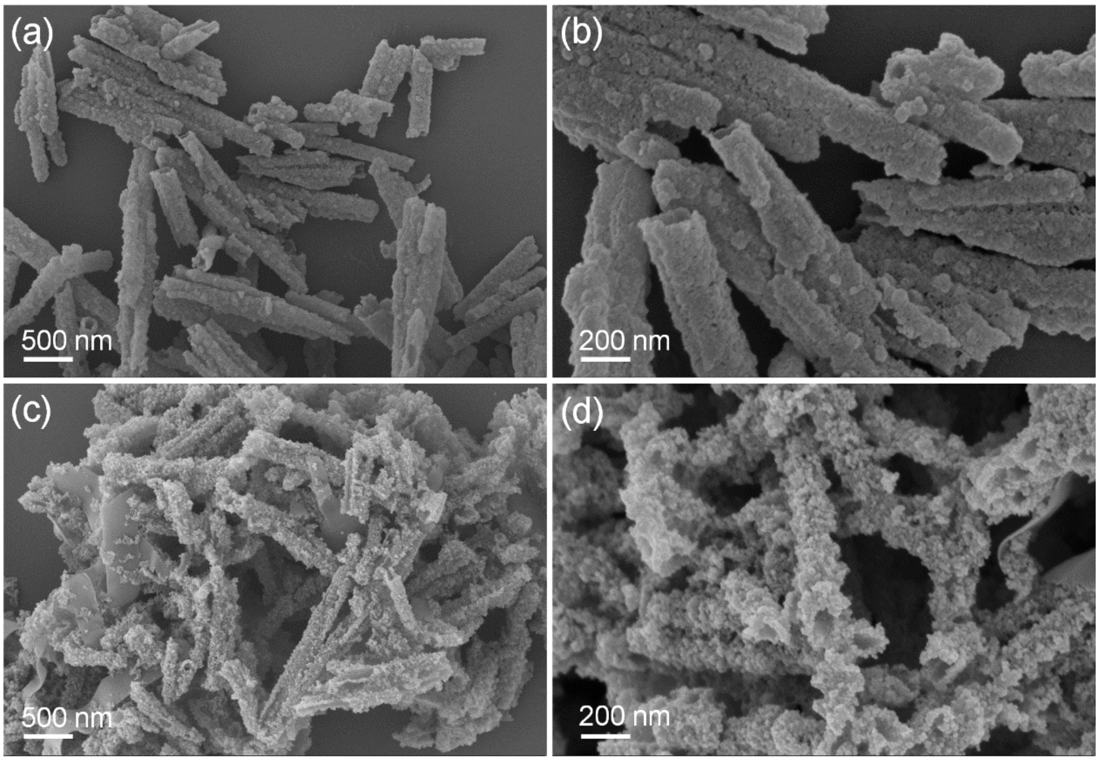

Figure S4. SEM of (a-b) $NiPO_x$/C, and (c-d) Ru-Ni_2P/C nanotubes at different magnifications.

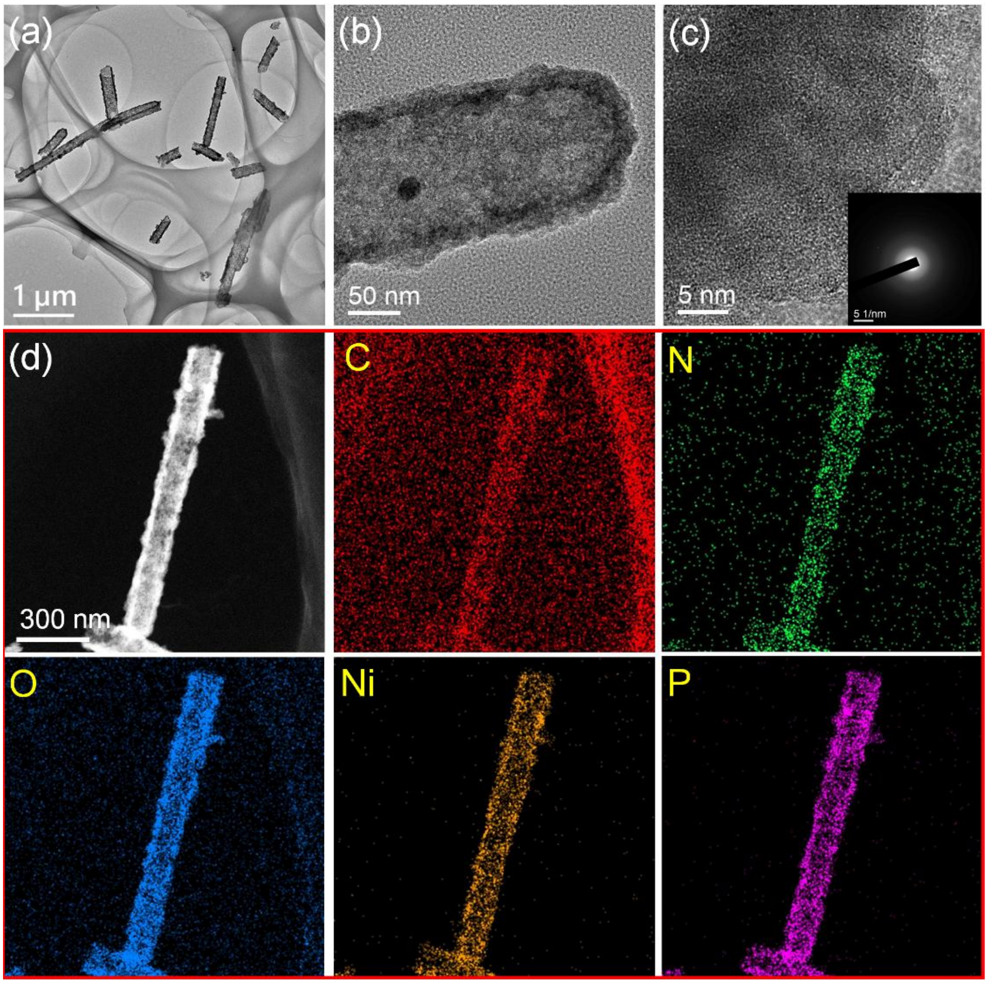

Figure S5. (a-b) TEM images, (c) HRTEM image, and SAED pattern (inset) of $NiPO_x/C$. (d) STEM image and the corresponding elemental mappings of $NiPO_x/C$.

The $NiPO_x/C$ catalysts were obtained by pyrolysis at 500 ◦C under an Ar atmosphere. The SEM and low-magnification TEM results (Figure S4 and S5) revealed that the original nanotube structure of the MOF precursors was well inherited. Interestingly, the amorphous nature of the nanotubes was maintained after the heat treatment. This observation was supported by the absence of lattice fringes in the high-resolution (HRTEM) image (Figure S5c) and the selected area electron diffraction (SAED) pattern (inset in Figure S5c) displaying a diffuse halo without distinct diffraction rings or spots. The corresponding HAADF-STEM image and elemental mapping analysis of $NiPO_x/C$ catalysts revealed the uniform distribution of Ni, P, C, N, and O in the nanotube structure (Figure S5d). According to the above results, the unique mesoporous structure can be indexed to an amorphous Ni-phosphate-embedded *N*-doped carbon ($NiPO_x/C$) matrix with well-inherited nanotube features.

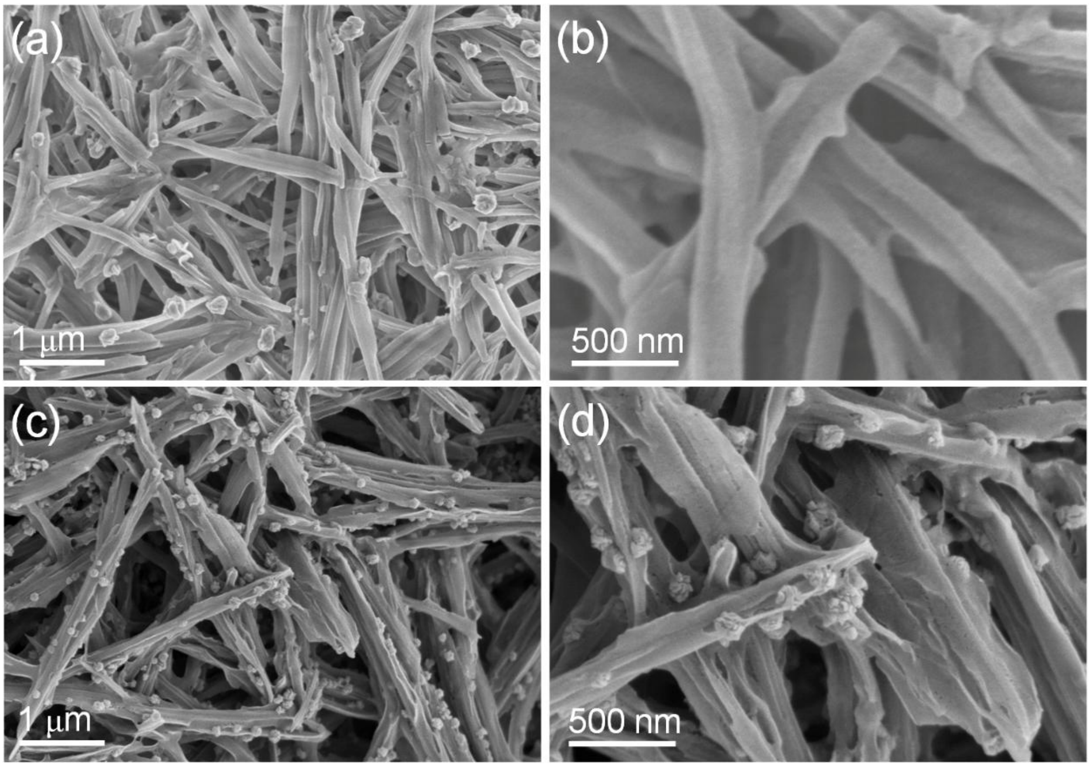

Figure S6. SEM of (a-b) Ni-BTC(S)/HOF, and (c-d) RuNi-BTC(S)/HOF nanotubes at different magnifications.

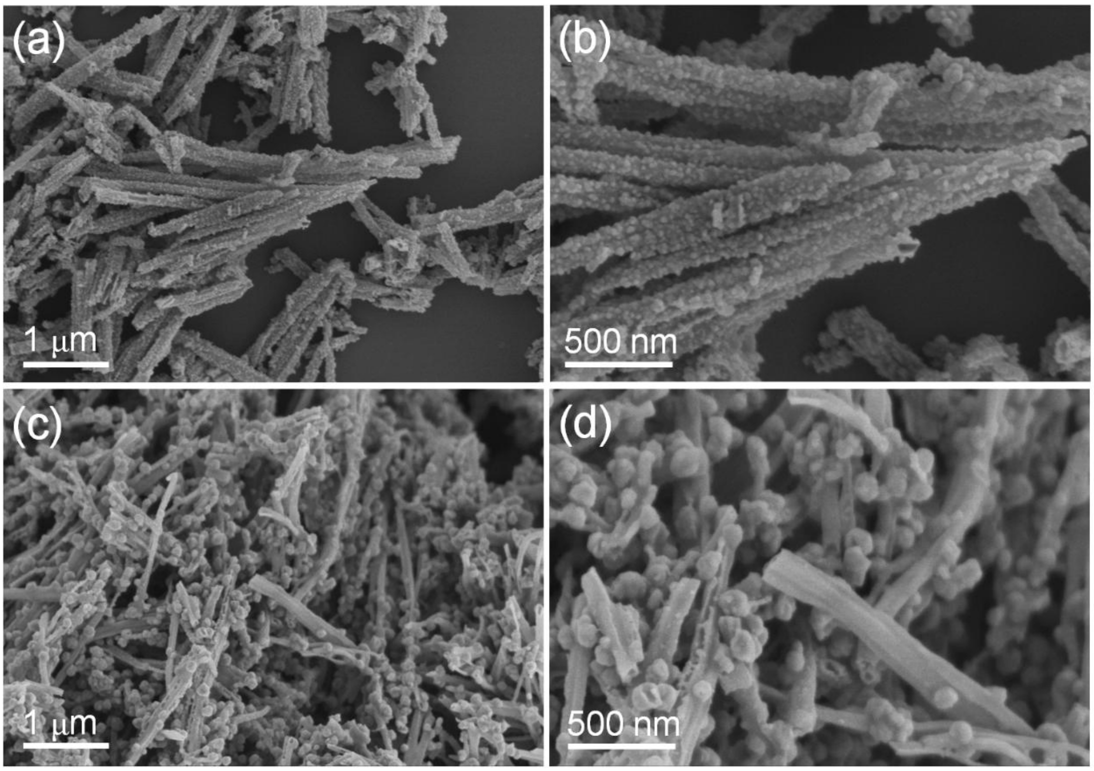

Figure S7. SEM of (a-b) NiS/Ni_9S_8/C, and (c-d) Ru-Ni_9Se_8/C nanotubes at different magnifications.

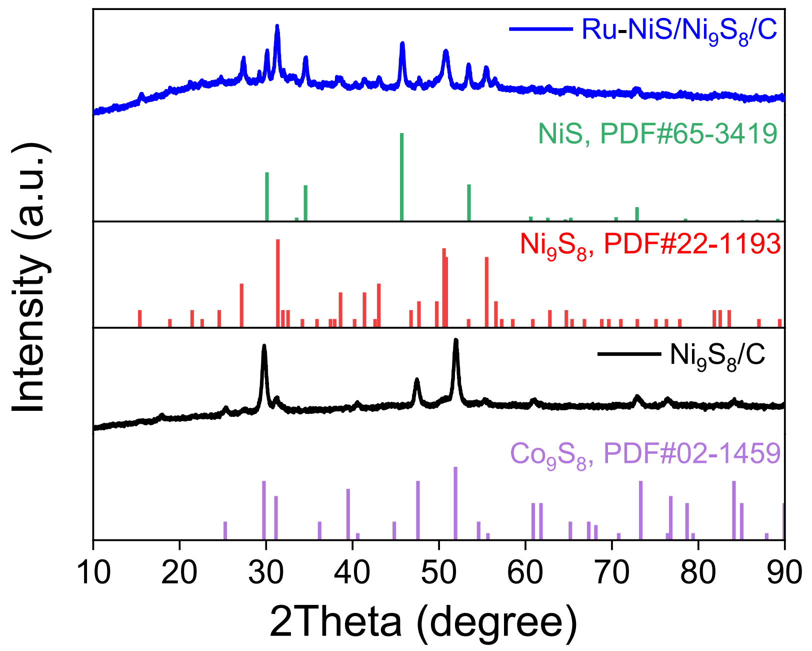

Figure S8. XRD patterns of NiS/Ni_9S_8/C and Ru-Ni_9S_8/C catalysts.

NiS/Ni_9S_8/C and Ru-Ni_9S_8/C nanotubes (Figure S6) were prepared using above mentioned solvothermal method, which exhibited typical one-dimensional (1D) tube-like structures with diameters of approximately 100 nm. After calcination under Ar gas, NiS/Ni_9S_8/C and Ru-Ni_9S_8/C nanotubes were formed (Figure S7 and S8). The rough surface of the nanotubes indicates the formation of massive nanoparticles on the shells.

The crystal structures of the catalysts were studied using XRD analysis. As illustrated in Figure S8, the phase compositions of the Ru-Ni_9S_8/C nanotubes were identified as a combination of NiS (JCPDS: 65−3419) and Ni_9S_8 (JCPDS: 22−1193). While the phase compositions of the Ru-Ni_9S_8/C nanotubes were identified as Ni_9S_8 (Co_9S_8, JCPDS: 02−1459).

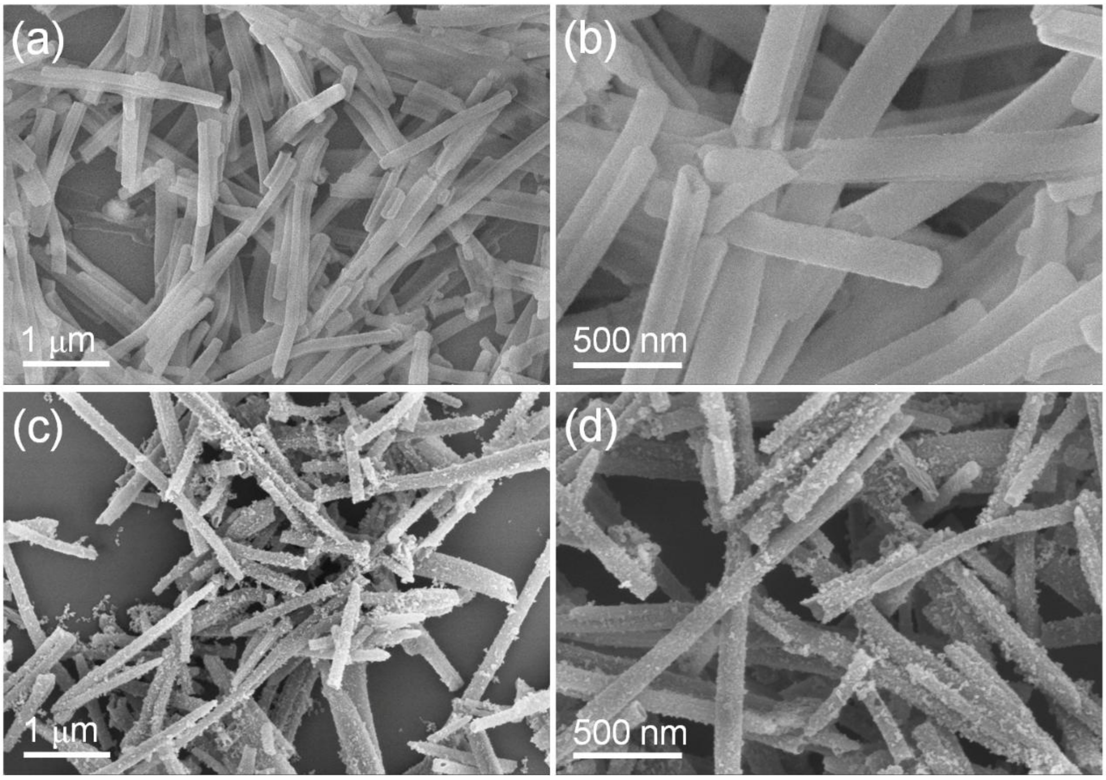

Figure S9. SEM of (a-b) Ni-BTC(Se)/HOF, and (c-d) RuNi-BTC(Se)/HOF nanotubes at different magnifications.

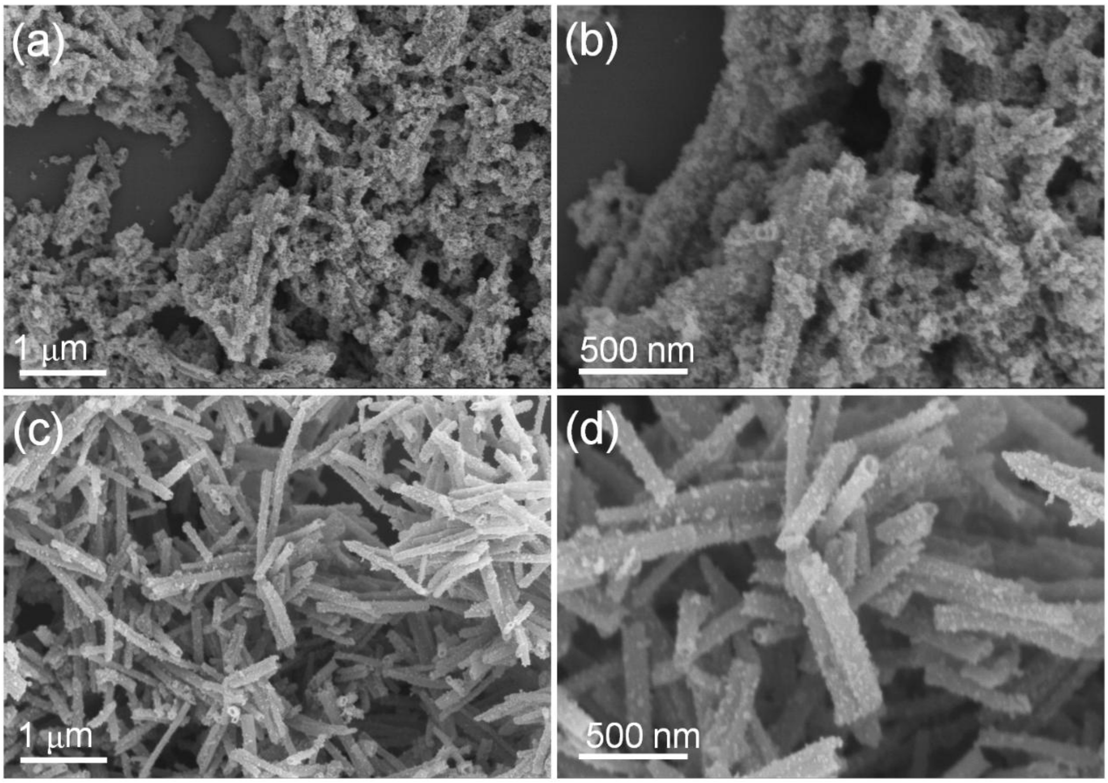

Figure S10. SEM of (a-b) Ni_3Se_4/C, and (c-d) Ru-Ni_3Se_4/C nanotubes at different magnifications.

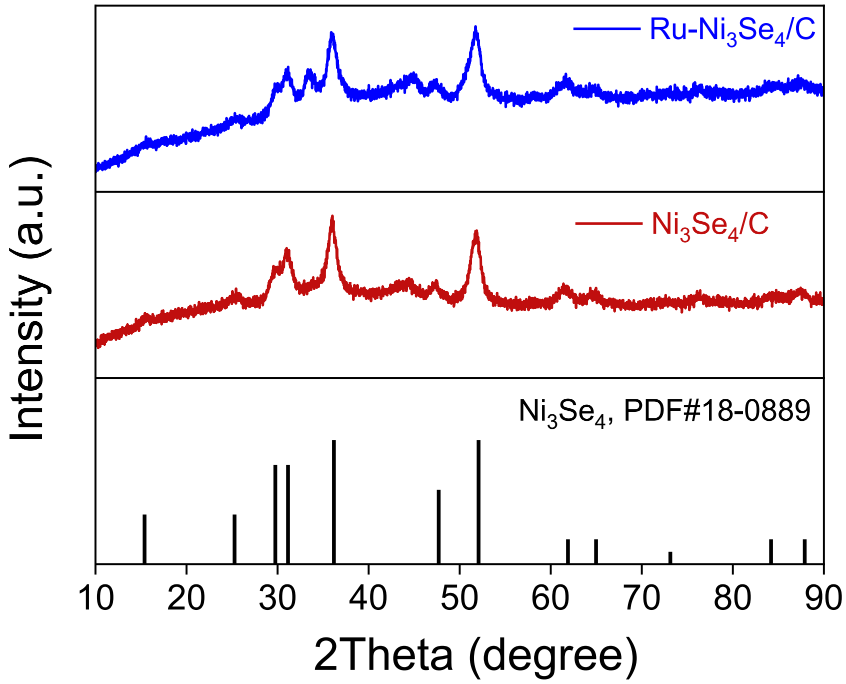

Figure S11. XRD patterns of Ni_3Se_4/C and Ru-Ni_3Se_4/C catalysts.

The Ni_3Se_4/C and Ru-Ni_3Se_4/C nanotubes (Figure S9) were also prepared using above mentioned solvothermal method, which exhibited typical one-dimensional (1D) tube-like structures with diameters of approximately 100 nm. After calcination under Ar gas, Ni_3Se_4/C and Ru-Ni_3Se_4/C nanotubes were formed (Figure S10 and S11). The rough surface of the nanotubes indicates the formation of massive nanoparticles on the shells. The XRD patterns of the catalysts derived from Ni-BTC(Se)/HOF and RuNi-BTC(Se)/HOF precursors can be indexed to Ni_3Se_4 (JCPDS: 18-0889), as depicted in Figure S11.

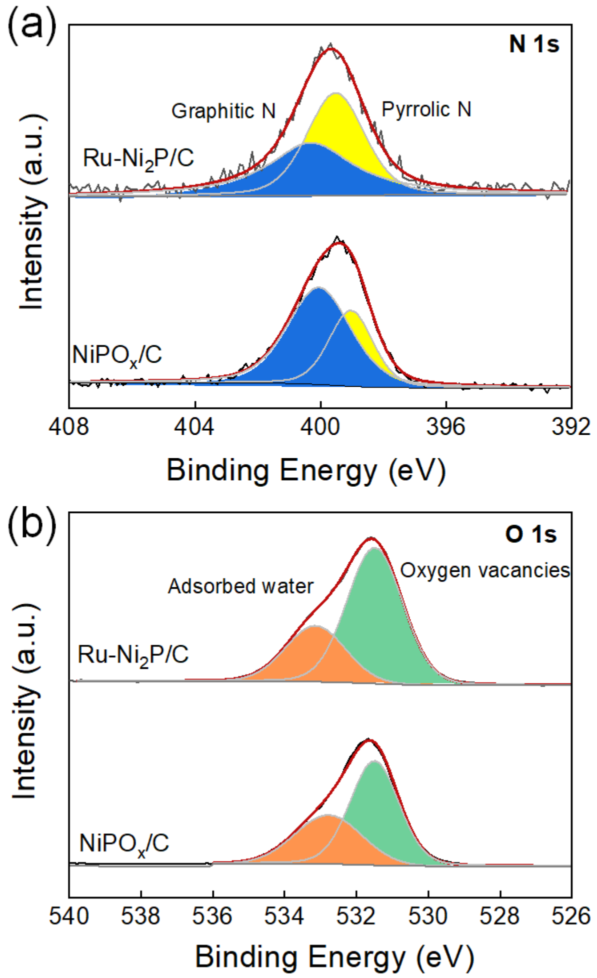

Figure S12. High-resolution XPS spectra corresponding to (a) N 1s, and (b) O 1s.

The N 1s spectrum are deconvoluted into two major peaks representing pyrrolic-N, and graphitic-N (Figure S12a). These peaks are likely derived from the triazine and amino groups inherent in melamine. The stronger graphitic-N peaks observed for Ru–Ni_2P/C compared with $NiPO_x/C$ suggest enhanced electrical conductivity. The O 1s spectrum (Figure S12b) is deconvoluted into two peaks, which could be assigned to O in adsorbed water (532.7 eV), and oxygen vacancies (531.3 eV), respectively.

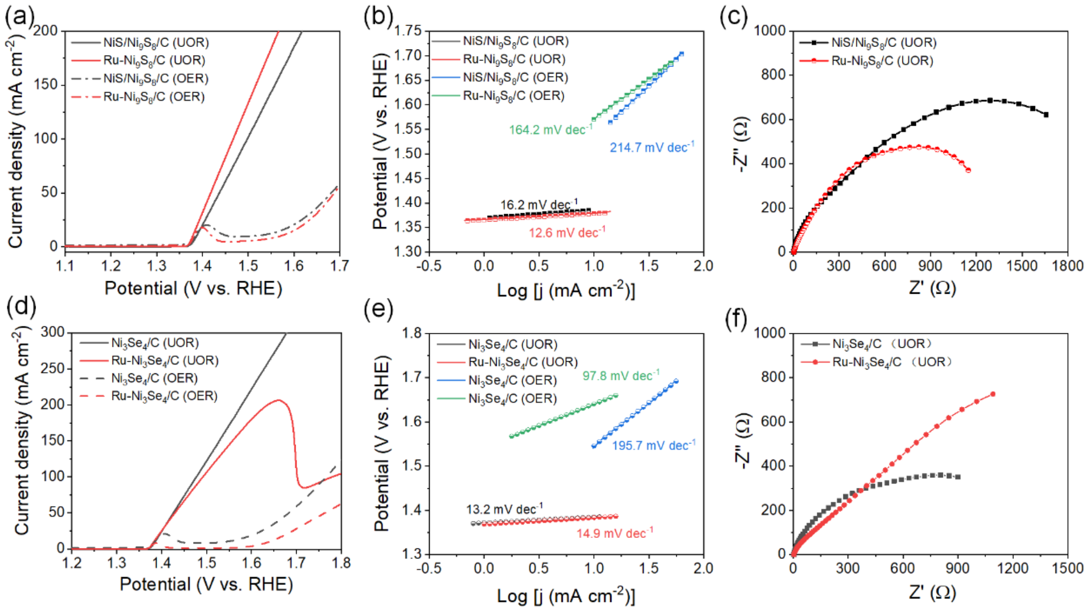

Figure S13. (a) LSV curves, (b) Tafel plots, and (c) Nyquist plots tested at 1.347 V (vs. RHE) of $NiS/Ni_9S_8/C$ and Ru–Ni_9S_8/C catalysts in 1M KOH aqueous electrolyte with and without 0.33 M urea; (d). (a) LSV curves, (b) Tafel plots, and (c) Nyquist plots tested at 1.347 V (vs. RHE) of Ni_3Se_4/C and Ru–Ni_3Se_4/C catalysts in 1M KOH aqueous electrolyte with and without 0.33 M urea;

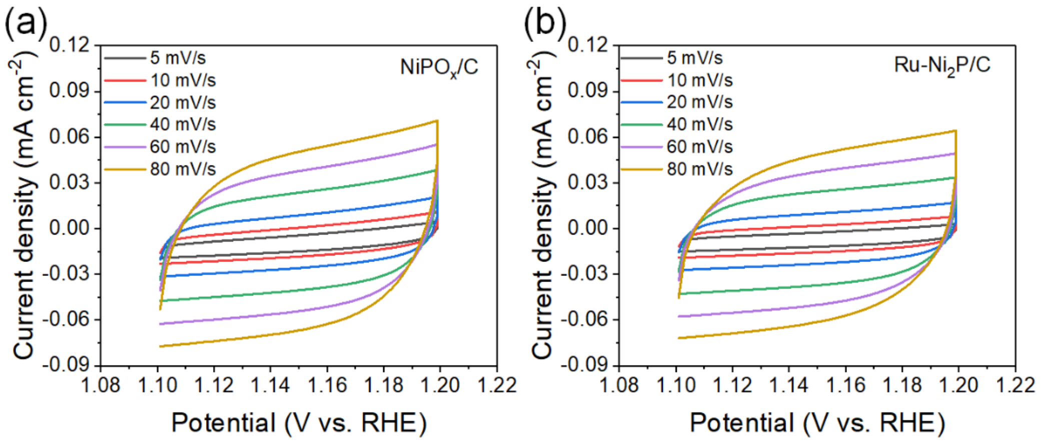

Figure S14. CV curves of (a) $NiPO_x/C$, (b) Ru-Ni_2P/C at different scan rates (5, 10, 20, 40, 60, and 80 mV/s).

Table S1. Comparison of catalytic performance of $NiPO_x/C$, $Ru-Ni_2P/C$ and some recently reported catalysts toward UOR.

**Catalysts**	**Electrolyte**	**Potential @ 10 mA cm^{-2} (V)**	**Tafel Slope (mV dec^{-1})**	**Ref.**
$Ru-Ni_2P/C$	1.0 M KOH + 0.33 M urea	1.36	12.2	This work
$NiPO_x/C$	1.0 M KOH + 0.33 M urea	1.37	22.7	This work
$NiCo_{11}$/CoFcDCA	1 M KOH + 0.5 M urea	1.39	53.2	*Fuel* **2026**, 405, 136602.
CeO_2/NiO/Ni	1 M KOH + 0.33 M urea	1.35	51.4	*J. Mater. Chem. A* **2026**; https://doi.org/10.1039/d6ta04641j
RhNi metallene	1 M KOH + 0.33 M urea	1.38	15.3	*J. Colloid. Interface Sci.* **2026**, 702, 138860
Ru-NiO/Co_3O_4/NF	1 M KOH + 0.5 M urea	1.36	21.4	*J. Colloid Interface Sci.* **2025**, 683, 600-611.
Ni-Mo_2C/CNFs	1 M KOH + 0.33 M urea	1.33	25.4	*Adv. Energy Mater.* **2025**, 15, e04064
Ru/Co_3O_4/C	1.0 M KOH + 0.33 M urea	1.45	137.5	*Int J Hydrogen Energ*, **2025**, 157, 150472
WC@$Co(OH)_2$-2	1.0 M KOH + 0.33 M urea	1.38	113.0	*Small Methods* **2025**, 9, e0118
Ni–Fe–Co LDH/Ni–S/NF	1 M KOH + 0.5 M urea	1.32	36.0	*J. Mater. Chem. A* **2025**, 13, 18851–18869.
Pt-$Ni(OH)_2$@Ni-CNFs	1 M KOH + 0.33 M urea	1.36	13.7	*Energy Environ. Sci.* 17(5), **2024**, 1984-1996
Rh/$LaNiO_3$	1 M KOH + 0.33 M urea	1.49	68.0	*Int. J. Hydrogen Energy* 56, **2024**, 16-26
Ni/MoC@NC	1 M KOH + 0.33 M urea	1.38	38.0	*Int. J. Hydrogen Energy* 60, **2024**, 46-54

ZIF-67/Ni(OH)$_2$	1 M KOH + 0.3 M urea	1.32	35.0	*Surf. Interfaces* **2024**, *48*, 104273.
Ni_2P	1.0 M KOH + 0.5 M urea	1.35	100.0	*Nanoscale*, **2021**, 13, 1759-1769.
Ni-MOF@NiO/Ni	1.0 M KOH + 0.33 M urea	1.40	48.1	*Chem. Eng. J.* **2021**, *417*, 129201.
Ru/B-Ni_2P/Ni_5P_4	1 M KOH + 0.33 M urea	1.37	43.8	*J. Mater. Chem. A* 10(30), **2022,** 16236-16242.
